# Knockdown of Sestrin2 Increases Lipopolysaccharide-Induced Oxidative Stress, Apoptosis, and Fibrotic Reactions in H9c2 Cells and Heart Tissues of Mice via an AMPK-Dependent Mechanism

**DOI:** 10.1155/2018/6209140

**Published:** 2018-07-05

**Authors:** Hwan-Jin Hwang, Joo Won Kim, Hye Soo Chung, Ji A. Seo, Sin Gon Kim, Nan Hee Kim, Kyung Mook Choi, Sei Hyun Baik, Hye Jin Yoo

**Affiliations:** Division of Endocrinology and Metabolism, Department of Internal Medicine, College of Medicine, Korea University, Seoul, Republic of Korea

## Abstract

Sestrin2 (sesn2) is an endogenous antioxidant protein that has recently gained attention for its potential to treat various inflammatory diseases. However, the relationship of sesn2 with cardiomyopathy is still unclear. In H9c2 cells, sesn2 knockdown reduced the level of 5′ adenosine monophosphate-activated protein kinase (AMPK) phosphorylation, downregulated antioxidant genes including catalase and superoxide dismutase (SOD2), and increased reactive oxygen species (ROS) production upon lipopolysaccharide (LPS) treatment. LPS-mediated cell death and the expression of matrix metalloproteinase (MMP) 2 and MMP9 were significantly increased by sesn2 knockdown. However, these increases were prevented by treatment with 5-aminoimidazole-4-carboxamide ribonucleotide (AICAR), an AMPK activator. Consistent with the *in vitro* results, AMPK phosphorylation was decreased in heart tissue from sesn2 knockdown mice compared to heart tissue from control C57BL/6 mice, which was associated with decreased expression of antioxidant genes and increased LPS-mediated cell death signaling. Furthermore, the decrease in AMPK phosphorylation caused by sesn2 knockdown increased LPS-mediated expression of cardiac fibrotic factors, including collagen type I and type III, in addition to MMP2 and MMP9, in heart tissue from C57BL/6 mice. These results suggest that sesn2 is a novel potential therapeutic target for cardiomyopathy under inflammatory conditions.

## 1. Introduction

Cardiomyopathy refers to any abnormality of the myocardium leading to a clinical condition in which the heart cannot deliver sufficient blood to the body. Left ventricular hypertrophy and reduction of ejection fraction, caused by cardiac remodeling involving the hypertrophy or apoptosis of cardiomyocytes and excessive deposition of collagen fibers in the extracellular matrix, are major features observed in patients with cardiomyopathy [1]. The onset and development of cardiomyopathy are triggered by a variety of risk factors such as inflammation, hyperlipidemia, and insulin resistance [2, 3].

High oxidative stress results in myocardial distortion, manifested by extracellular matrix remodeling, myocyte apoptosis, and interstitial fibrosis [2, 4]. Wang et al. [5] demonstrated that treatment with hydrogen peroxide (H_2_O_2_), an inducer of oxidative stress, upregulates collagen expression in cardiac fibroblasts. Matrix metalloproteinases (MMPs), which are zinc-dependent endopeptidases, were originally identified as collagen matrix remodeling factors. There are 25 different MMPs. MMP2 and MMP9 are activated by reactive oxygen species- (ROS-) mediated inflammatory signaling [6] and are involved in cardiovascular diseases [7]. Under oxidative conditions, MMP2 has been shown to significantly induce cardiomyocyte apoptosis [8]; moreover, deletion of the MMP9 gene in cardiac muscle recovered the ejection fraction of the left ventricle by reducing macrophage infiltration and fibrosis [9]. Therefore, the inhibition of excessive production of ROS-induced MMPs might be an important step to protect cardiac muscle from apoptosis and fibrotic reactions, which can lead to cardiomyopathy [7, 10].

Sestrin (sesn) was recently identified as a novel antioxidant molecule whose expression is upregulated in cells exposed to various stresses, including hypoxia and oxidative stimuli [11]. In mammals, three sesns (sesn1–3) have been characterized. Sesn2 negatively regulates the mammalian target of rapamycin (mTOR) signaling by activating 5′ adenosine monophosphate-activated protein kinase (AMPK) and tuberous sclerosis complex 2 (TSC2) phosphorylation [12]. Interactions between sesn2 and the AMPK pathway have been shown to play a crucial role in the regulation of energy homeostasis, cell growth, and apoptosis [13, 14]. Park et al. [15] reported that obesity-induced hepatic endoplasmic reticulum (ER) stress and apoptosis were elevated in sesn2-deficient mice compared to normal mice. In vascular endothelial cells, inhibition of sesn2 was shown to elevate ROS production and cytotoxicity induced by inflammatory stimuli [16, 17]. Although growing evidence suggests that sesn2 protects against various cardiometabolic diseases such as nonalcoholic fatty liver disease (NAFLD) and atherosclerosis, it is unclear whether sesn2 has a beneficial effect against cardiomyopathy-related molecular events.

Toll-like receptor 4 (TLR4) is strongly involved with myocardium abnormality. The treatment of short hairpin RNA (shRNA) for TLR4 decreased inflammatory cytokine production, fibrotic area, and left ventricle infarct size and recovered fractional shortening of the left ventricle in a rat myocardial infarction (MI) model [18]. In human, cardiac TLR4 levels were elevated in patients with dilated cardiomyopathy [19]. Increased circulating levels of lipopolysaccharide (LPS), a TLR4 agonist, were observed in patients with type 2 diabetes and decompensated heart failure, which are clinical conditions associated with cardiomyopathy [20, 21]. These data suggest that TLR4-mediating signaling is important to regulate the function of the heart. Therefore, we focused on the function of sesn2 against LPS treatment using H9C2 cells and heart tissue of C57BL/6 mice.

Therefore, to clarify whether the antioxidative effects of sesn2 were protective against LPS treatment, we examined (i) whether sesn2 knockdown decreased AMPK phosphorylation, (ii) whether sesn2 knockdown regulated ROS production and antioxidant gene expression, (iii) whether sesn2 knockdown regulated the expression of apoptosis-related molecules and cardiomyocyte death, and (iv) whether sesn2 knockdown increased cardiomyopathy-related factors (e.g., MMP2, MMP9, collagen I, and collagen III) upon LPS-induced inflammation in H9c2 rat cardiomyocytes and heart tissue obtained from C57BL/6 mice.

## 2. Materials and Methods

### 2.1. Cell Culture, Transfection, and Reagents

H9c2 cells (Korean Cell Line Bank, Seoul, South Korea) were maintained in Dulbecco's modified Eagle's medium (DMEM) (Invitrogen, Carlsbad, CA, USA) containing 10% (*v*/*v*) fetal bovine serum (FBS) (Invitrogen), 50 U/ml penicillin, and 50 g/ml streptomycin (Invitrogen). Cells were propagated at 37°C with 5% CO_2_ and subcultured in 24-well plates until reaching 90% confluency, after which they were transfected with rat sesn2-targeting siRNA or scrambled siRNA (Genolution, Seoul, South Korea) using Lipofectamine™ according to the user manual (Invitrogen). After 1 day, the efficiency of sesn2 knockdown was determined by Western blotting. LPS (Sigma-Aldrich, MO, USA), 5-aminoimidazole-4-carboxamide ribonucleotide (AICAR) (Sigma-Aldrich), and N-acetyl-cysteine (NAC) (Sigma-Aldrich) were dissolved in phosphate-buffered saline (PBS) (Biosesang, Seoul, Korea) and used to treat the transfected H9c2 cells. All additives were cotreated; cells were not serum starved.

### 2.2. Animals

Five-week-old male C57BL/6 mice purchased from SLC (Shizuoka, Japan) were randomly divided into the following 4 groups: scramble siRNA treatment (C), scramble siRNA plus LPS (50 *μ*g per mouse) treatment (L), sesn2-targeting siRNA (60 *μ*g per mouse) plus LPS treatment (LS), and sesn2-targeting siRNA plus LPS with AICAR (LSA) (AMPK activator; 12.5 mg per mouse). Sesn2-targeting siRNA was delivered into heart tissue using the in vivo jetPEI™ reagent (Polyplus-transfection, New York, USA) according to the user manual. LPS and AICAR were administered to mice by intraperitoneal injection. After 1 day, heart tissue was harvested. A detailed experimental design is described in our previous report [17]. This study was approved by the Institutional Animal Care and Use Committee (IACUC) of Korea University (Seoul, Korea).

### 2.3. Western Blotting

Total protein extracts of H9c2 cells and heart tissue were generated using PRO-PREPTM (iNtRON, Sungnam, Korea). Proteins were separated by sodium dodecyl sulfate polyacrylamide gel electrophoresis (SDS-PAGE). The separated proteins were transferred to 0.45 *μ*m nitrocellulose membranes (Amersham Bioscience, Westborough, MA, USA). The membranes were incubated sequentially with blocking solution (0.05% TBST containing 5% nonfat dry milk or 5% bovine serum albumin), blocking solution plus primary antibodies, and blocking solution plus horseradish peroxidase-conjugated secondary antibodies (Amersham Bioscience). The membranes were incubated with chemiluminescence solution (Bio-Rad) to detect immunoreactive bands in the dark. The following antibodies were used: anti-beta actin mouse monoclonal IgG (1 : 5000 dilution), anti-beta actin rabbit polyclonal IgG (1 : 1000 dilution), anti-sesn2 mouse monoclonal IgG (1 : 500 dilution), anti-catalase mouse monoclonal IgG (1 : 1000 dilution), anti-superoxide dismutase 2 (SOD2) mouse monoclonal IgG (1 : 500 dilution), anti-MMP2 mouse monoclonal IgG (1 : 1000 dilution), anti-MMP9 mouse monoclonal IgG (1 : 1000 dilution), anti-collagen type I alpha I mouse monoclonal (1 : 1000 dilution), and anti-collagen type III alpha I mouse monoclonal IgG (1 : 1000 dilution; Santa Cruz Biotechnology, Santa Cruz, CA, USA). In addition, anti-pAMPK rabbit monoclonal (1 : 1000 dilution), anti-total AMPK rabbit monoclonal (1 : 2000 dilution), anti-Bcl2-associated X protein (Bax) rabbit polyclonal (1 : 1000 dilution), and anti-B-cell lymphoma-extra large (Bcl-xL) rabbit polyclonal antibodies (1 : 1000 dilution; Cell Signaling Technology, Boston, MA, USA) were used.

### 2.4. Measurement of ROS Production

H9c2 cells were stained with 10 *μ*M dihydroethidium (DHE) red (Invitrogen) for 60 min at 37°C to detect ROS levels. Stained cells were then fixed in 10% formalin for 10 min and incubated with 4′,6-diamidino-2-phenylindole (DAPI) blue (Sigma-Aldrich) for nuclear staining. The stained cells were observed under a fluorescence microscope (Olympus, Japan). Staining was quantified using ImageJ software (National Institutes of Health, Bethesda, MD, USA).

### 2.5. Hoechst Staining

To observe the shapes of their nuclei, H9c2 cells were incubated with 1 *μ*g/ml Hoechst dye (blue) (Sigma-Aldrich) for 30 min at room temperature. Nuclear degradation was then assessed under a fluorescence microscope from the 4 fields of view randomly selected in each well, and then representative images were obtained for each group.

### 2.6. Terminal Deoxynucleotidyl Transferase dUTP Nick End Labeling (TUNEL) Assay

ApoBrdU DNA Fragmentation Assay Kit (Biovision, Milpitas, CA, USA) was used to detect DNA damage according to the user manual. TUNEL-negative cells (only blue) and positive cells (blue plus green) were counted under a fluorescence microscope from the 4 fields of view randomly selected in each well, and then representative images were obtained for each group.

### 2.7. Measurement of Cell Viability and Cytotoxicity

H9c2 cell viability was measured using an EZ-CYTOX kit (DAEILAP, Seoul, Korea) and LDH (lactate dehydrogenase) cytotoxicity assay kit (DAEILAP) according to the user manual. Cells were incubated with EZ-CYTOX solution or LDH assay solution for 30 min, after which the optical density (OD) was measured using a microplate reader (Bio-Rad).

### 2.8. Caspase-3 Activity Assay

Heart tissue samples were homogenized with lysis buffer and their total protein contents were quantified using the Bradford method. Caspase-3 activity was quantitated in equal protein amounts of tissue extract using a Caspase-3 Assay Kit (Abcam, MA, USA) according to the user manual. The OD value at 400 nm was measured using a microplate reader.

### 2.9. Measurement of Antioxidants and Gelatinase Activities

Total protein extracted from heart tissues was used for measurement of catalase, SOD, and gelatinase activities. Their activities were analyzed in equal protein amounts using EZ-Catalase Assay Kit (DAEILAP), EZ-SOD Assay Kit (DAEILAP), and Gelatinase Assay Kit (Biovision) according to the user manual.

### 2.10. Statistical Analysis

All statistical analyses were performed using SPSS for Windows (version 12.0; SPSS Inc., Chicago, IL, USA). The significance of differences between groups was determined by analysis of variance (ANOVA). All graphs present data as means ± SDs from more than three experiments. Differences were considered to be significant at *P* < 0.05.

## 3. Results

### 3.1. Sesn2 Knockdown Increases LPS-Induced Oxidative Stress in H9c2 Cells

**Figure 1:**
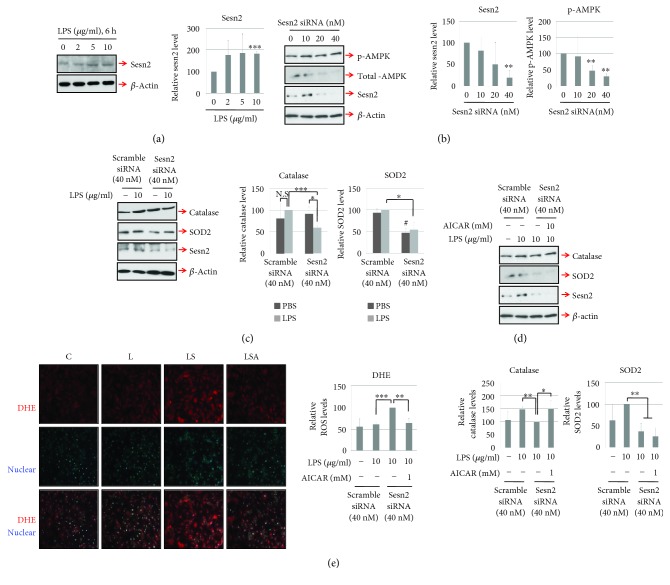
Sesn2 knockdown increases LPS-mediated oxidative stress through an AMPK-dependent pathway in H9c2 cells. (a) H9c2 cells were stimulated with various doses of LPS for 6 h. (b) siRNA targeting rat sesn2 was transfected into H9c2 cells, and the level of phosphorylated AMPK was determined by Western blotting. (c and d) Sesn2 knockdown H9c2 cells were incubated with LPS or LPS plus AICAR for 4 h. Western blotting was then used to determine the levels of catalase and SOD2. (e) Sesn2 knockdown cells were stimulated with LPS or LPS plus AICAR for 6 h, after which ROS levels were analyzed by DHE staining (red). All graphs were obtained from three separate experiments. Data are presented as means; error bars represent ±SD (NS: not significant; ^#^*P* < 0.05 versus control; ^∗^*P* < 0.05; ^∗∗^*P* < 0.005; ^∗∗∗^*P* < 0.0005; ANOVA).

In H9c2 cells, the expression of Sesn2 was significantly increased under the treatment of LPS (Figure 1(a)). To determine the function of sesn2 in heart muscle cells, siRNA directed against sens2 was transfected into H9c2 cells. Western blotting showed that AMPK phosphorylation was decreased in sesn2 knockdown cells (Figure 1(b)). The knockdown of sesn2 in cardiomyocytes was associated with the expression of various antioxidant genes, including catalase and SOD2. In sesn2 knockdown H9c2 cells, the catalase level was significantly decreased after LPS treatment; however, this decrease was prevented by treatment with AICAR, an AMPK activator (Figures 1(c) and 1(d)). The level of SOD2 was similarly decreased by sesn2 knockdown in H9c2 cells with or without LPS treatment; however, this decrease was not prevented by AICAR treatment (Figures 1(c) and 1(d)). Furthermore, LPS-mediated ROS production, as measured by DHE staining, was significantly increased in sesn2 knockdown H9c2 cells. This increase was prevented in sesn2 knockdown H9c2 cells treated with AICAR (Figure 1(e)).

### 3.2. Sesn2 Knockdown Increases LPS-Mediated Death in H9c2 Cells

**Figure 2:**
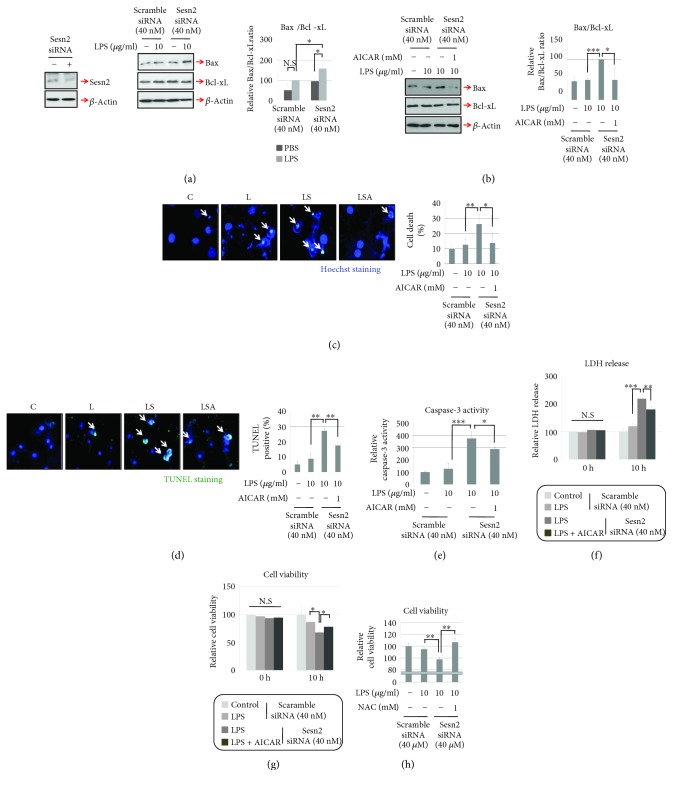
H9c2 cell viability is decreased by sesn2 knockdown upon LPS treatment. (a and b) Sesn2 knockdown H9c2 cells were incubated with LPS or LPS plus AICAR for 6 h, after which the ratio of Bax to Bcl-xL was determined by Western blotting. (c) Sesn2 knockdown cells were stimulated with LPS or LPS plus AICAR for 10 h, after which nuclei were observed by Hoechst staining (blue). (d) TUNEL-negative cells (only blue) and TUNEL-positive cells (blue plus green) were counted under a fluorescence microscope from the 4 fields of view randomly selected in each well, and then representative images were obtained for each group. (e) Caspase-3 activity in H9c2 cells was measured using a Caspase-3 Activity Assay Kit. (f) LDH release and (g) cell viabilities were calculated using a LDH assay reagent and cell viability assay reagent. (h) Sesn2 knockdown H9c2 cells were incubated with LPS or LPS plus NAC for 10 h, after which cell viabilities were measured. All graphs were obtained from more than three independent experiments. Data are presented as means; error bars represent ±SD (NS: not significant; ^∗^*P* < 0.05; ^∗∗^*P* < 0.005; ^∗∗∗^*P* < 0.0005; ANOVA).

We hypothesized that the increased ROS level in sesn2 knockdown cells might reduce their viability. With the goal of assessing proapoptotic events in sesn2 knockdown cells, we quantified the ratio of the proapoptotic molecule Bax to the antiapoptotic molecule Bcl-xL by Western blotting. In sesn2 knockdown H9c2 cells, LPS treatment increased the Bax/Bcl-xL ratio; AICAR treatment completely prevented this increase (Figures 2(a) and 2(b)). In addition, LPS-induced nuclear degradation, as measured by Hoechst staining, and fragmentation, as detected by TUNEL staining, and caspase-3 activity were significantly increased in sesn2 knockdown cells. Similar to the Western blotting results, this increase was attenuated by AICAR treatment (Figures 2(c)–2(e)). Finally, we measured cell viability and cytotoxicity. While LPS treatment alone did not affect LDH release in H9c2 cells, sesn2 knockdown cells treated with LPS exhibited increased LDH release. However, LDH release was recovered by AICAR treatment (Figure 2(f)). Likewise, cell viabilities were significantly decreased in sesn2 knockdown cells treated with LPS, but these reductions disappeared after the treatment AICAR or N-acetyl-cysteine (NAC), an antioxidant (Figures 2(g) and 2(h)).

### 3.3. Sesn2 Knockdown Increases Production of LPS-Induced Cardiac Matrix Metalloproteinases in H9c2 Cells

**Figure 3:**
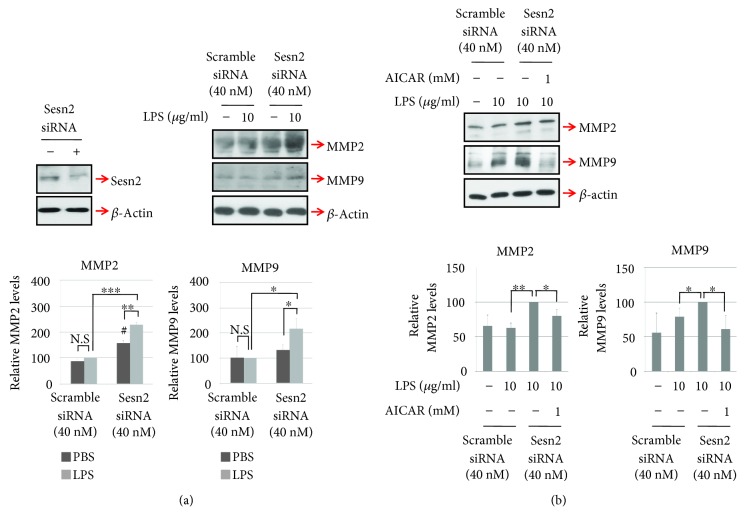
LPS-induced expression of MMP2 and MMP9 is increased by sesn2 knockdown in H9c2 cells. (a and b) Sesn2 knockdown cells were stimulated with LPS or LPS plus AICAR for 6 h, after which Western blotting was performed to assess the levels of MMP2 and MMP9. All graphs were obtained from three independent experiments. Data are presented as means; error bars represent ±SD (NS: not significant; ^#^*P* < 0.05 versus control; ^∗^*P* < 0.05; ^∗∗^*P* < 0.005; ^∗∗∗^*P* < 0.0005; ANOVA).

Western blot analysis showed that MMP2 and MMP9 were regulated by the intracellular sesn2 level. Under without LPS treatment, sens2 knockdown increased the expression of MMP2, but did not affect the MMP9 level (Figure 3(a)). MMP2 and MMP9 were expressed at higher levels in sesn2 knockdown H9c2 cells compared to H9c2 cells upon LPS treatment (Figure 3(a)). However, AICAR treatment decreased LPS-mediated upregulation of MMP2 and MMP9 expression in sesn2 knockdown H9c2 cells (Figure 3(b)).

### 3.4. Sesn2 Knockdown Increases Oxidative and Apoptotic Reactions to LPS in Heart Tissue from C57BL/6 Mice via an AMPK-Dependent Mechanism

**Figure 4:**
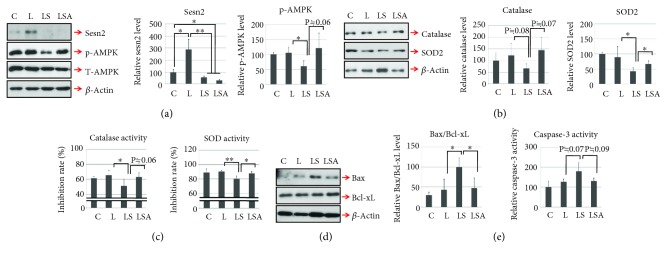
Sesn2 knockdown increases LPS-mediated cell death signaling in C57BL/6 mice. (a) To assess sens2 knockdown efficiency in heart tissue, Western blotting was used to measure the protein levels of sesn2 and phosphorylated AMPK. (b) The levels of catalase and SOD2 were determined by Western blotting. (c) The activities of catalase and SOD were measured using commercial assay kit. (d) The ratio of Bax to Bcl-xL was detected by Western blotting. (e) Caspase-3 activity in heart tissue was measured using a Caspase-3 Activity Assay Kit. All graphs present data from four mice per group (C: control mice; L: LPS only-treated mice; LS: sesn2 siRNA plus LPS-treated mice; LSA: sesn2 siRNA plus LPS and AICAR-treated mice). Data are presented as means; error bars represent ±SD (^∗^*P* < 0.05; ^∗∗^*P* < 0.005; ANOVA).

**Figure 5:**
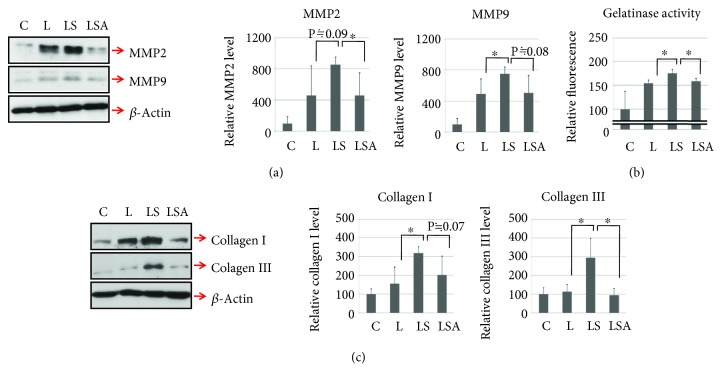
Cardiac matrix metalloproteinase and fibrotic factors are highly expressed in heart tissue from sesn2 knockdown mice after LPS treatment. (a) The levels of cardiac MMP2 and MMP9 were analyzed by Western blotting. (b) Cardiac gelatinase activity was measured using Gelatinase Assay Kit. (c) Fibrotic factors (collagen type I and collagen type III) were determined by Western blotting. (C: control mice; L: LPS only-treated mice; LS: sesn2 siRNA plus LPS-treated mice; LSA: sesn2 siRNA plus LPS and AICAR-treated mice). Data are presented as means; error bars represent ±SD (^∗^*P* < 0.05; ANOVA).

To examine the function of sesn2 *in vivo*, sesn2 expression was silenced by siRNA transfection. Consistent with the *in vitro* results, sesn2 knockdown was associated with decreased AMPK phosphorylation (Figure 4(a)). Furthermore, in the sesn2 knockdown LPS treatment group, the expression and activity of antioxidant molecules such as catalase and SOD2 (Figures 4(b) and 4(c)) were decreased and cell death-related signaling, demonstrated by the Bax/Bcl-xL ratio (Figure 4(d)) and caspase-3 activity (Figure 4(e)), was increased. However, these effects tended to disappear after the treatment with AICAR. Furthermore, sesn2 knockdown upregulated the expression of MMP2, MMP9, and cardiac fibrotic factors (collagen type I and III) in addition to cardiac gelatinase activity (Figures 5(a)–5(c)). Likewise, these effects were prevented by AICAR treatment (Figures 5(a)–5(c)).

## 4. Discussion

In H9c2 cells and mouse heart tissue, we found that (i) sesn2 knockdown reduced AMPK phosphorylation, (ii) sesn2 knockdown increased LPS-mediated ROS production by inhibiting the expression of antioxidant genes, (iii) sesn2 knockdown increased LPS-mediated apoptotic events, as demonstrated by the increased Bax/Bcl-xL ratio and enhanced nuclear degradation, and (iv) sesn2 knockdown upregulated LPS-mediated expression of MMP-2 and MMP-9. Under LPS treatment, (v) fibrotic factors such as collagen type I and collagen type III were highly expressed in sens2 knockdown heart tissue. Most of these oxidative, proapoptotic, and fibrotic effects in sesn2 knockdown H9c2 cells and mouse heart tissue were prevented by treatment with an AMPK activator, suggesting that sesn2 blocked LPS-mediated molecular events related to cardiomyopathy via an AMPK-dependent manner.

AMPK is a representative cardioprotective molecule. Deficiency of the AMPK*β* subunit or LKB1, a major AMPK upstream kinase, has been linked to impaired cardiac function in mice [22, 23]. Moreover, inactivation of the AMPK*α* subunit was shown to increase ischemic/reperfusion-mediated myocardial injury and apoptosis in mice [24]. Recently, sesn2 has been redefined as a novel regulator of AMPK signaling. Inhibition of sesn2 expression was shown to result in reduced AMPK phosphorylation in several tissue types [15, 17], whereas sesn2 overexpression has been shown to stimulate AMPK activity [12]. Morrison et al. [25] reported that sesn2 is a scaffold protein for the LKB1-AMPK axis; moreover, in heart tissue from sesn2 knockout mice, LKB1-mediated AMPK phosphorylation did not occur. This effect was shown to be related to the extent of myocardial infarct increase. Quan et al. [26] reported that the protein level of sesn2 was significantly decreased in heart tissue from older C57BL/6J mice; this effect was associated with left ventricular dysfunction under ischemic conditions. Furthermore, Dong et al. [27] showed that overexpression of sesn2 decreased phenylephrine-induced cardiomyocyte hypertrophy. Taken together, these data indicate that sesn2 directly influences the progression of cardiomyopathy by modulating the AMPK pathway. However, neither the effects of sesn2 on oxidative stress, apoptosis, and fibrotic reactions in heart tissues, which are the main pathologic features of cardiomyopathy, have not been explored nor their molecular mechanisms have been defined.

Antioxidant enzymes are required to remove excessive ROS, a major cause of cardiac dysfunction. In diabetic mice, overexpression of catalase (an antioxidant enzyme that converts hydroperoxide into oxygen and water) has been shown to reduce cardiac apoptosis and left ventricular abnormality by suppressing the ROS nuclear factor kappa B (NF*κ*B) axis [28]. Moreover, deficiency of SOD2, a mitochondrial antioxidant enzyme that converts superoxide to hydroperoxide, has been shown to result in enlarged hearts and left ventricular dilation in mice [29]. These antioxidant genes are regulated by peroxisome proliferator-activated receptor gamma coactivator 1-alpha (PGC-1*α*), a molecule downstream of AMPK [30, 31]. Considering the critical role of sesn2 in AMPK modulation, sesn2 may intimately regulate the expression of antioxidative genes in cardiomyocytes. In a cerebral ischemic/reperfusion model, sesn2 silencing was shown to increase brain infarct volume and downregulate the expression of two antioxidant genes, SOD2 and uncoupling protein 2 (UCP2), by inhibiting AMPK-PGC1*α* signaling [32]. Likewise, the present study demonstrated that sesn2 knockdown decreased the expression of catalase and SOD2 in H9c2 cells and mouse heart tissue. This resulted in increased ROS production, whereas AICAR treatment significantly prevented this effect. Therefore, the sesn2-AMPK axis appears to have an antioxidant effect in mouse heart tissues by regulating the expression of catalase and SOD2. Similar to our results, Seo et al. [33] showed that hepatic mitochondrial dysfunction, a leading cause of ROS production, was ameliorated by overexpression of sesn2 under glucose deprivation. However, unlike our expectations, the decreased SOD2 levels by sesn2knockdown were not improved after AICAR treatment in h9c2 cells. Although the exact underlying molecular mechanism about this could not be clarified in the present study, the relationship between the SOD2 expression and the sesn2-mediated AMPK action observed in heart tissues might be indirect and influenced by other factors.

Sesn2 is an important modulator of cell death. Previously, we reported that LPS treatment significantly reduced the viability of sesn2 knockdown human umbilical vein endothelial cells (HUVECs) [17]. Eid et al. [34] reported that sesn2-mediated AMPK activation inhibits enhanced ROS production in response to high glucose, thereby attenuating mesangial cell apoptosis. Ishihara et al. [35] showed that apoptosis of renal tubular cells incubated under hypoxic conditions was significantly reduced by overexpression of sesn2. In accordance with these studies, we showed that silencing sesn2 expression in LPS-treated H9c2 cells resulted in an elevated Bax/Bcl-xL ratio and reduced cell viability; however, these effects were prevented by treatment with an AMPK activator. Increased Bax/Bcl-xL ratios were observed in patients with ischemic heart disease and dilated cardiomyopathy [36]. Cardiac fibrosis, defined as the excessive accumulation of extracellular matrix components such as collagens, is a major pathologic feature of cardiomyopathy [37]. Spinale [1] proposed that myocardial remodeling refers to structural changes in the myocardium induced by matrix proteases, which result in left ventricular dysfunction. Increased levels of matrix proteases such as MMP2 and MMP9 were observed in heart tissue obtained from a patient with dilated cardiomyopathy. Furthermore, the cardiac content of collagen was shown to be increased in a patient with dilated cardiomyopathy, which was associated with increased myocardial stiffness in the left ventricle chamber [38, 39]. Previously, Zeng et al. [40] reported that sesn2 knockout mice exhibited increased cardiac fibrosis after irradiation injury, but the underlying molecular mechanism was not investigated. In the present study, inhibition of the sesn2-AMPK axis was related to increased expression of myocardial MMP2 and MMP9 upon LPS treatment. Furthermore, cardiac fibrotic factors such as collagen type I and type III were expressed more highly in sesn2 knockdown heart tissue than wild-type mouse heart tissue after LPS treatment. However, the increased collagen type I and type III found in heart tissues may be the results of replacement after cardiac cell death and not a directed event induced by sesn2 knockdown, because the expression of both collagens was not regulated by sesn2 knockdown in H9c2 cells (data not shown). Further studies should be followed to clarify the role of sesn2 as an endogenous molecule for potentially inhibiting myocardial fibrosis.

Sesn2 has been considered as a stress-inducible protein to maintain cellular homeostasis against several stimuli. The cells exposed to metabolic stress enhanced intracellular sesn2 levels to protect themselves [41]. Regulation of sesn2 levels is a defense mechanism observed in various cells and tissues. In our data, the increased sesn2 level after LPS treatment was associated with cellular protective effects in H9c2 cells and heart tissues. LPS-mediated oxidative, apoptotic, and fibrotic events were found in the sesn2 knockdown group, but not in the normal group. We thought that the increased sesn2 expression after LPS treatment led to protective effects against cardiomyopathy-related molecular events in the normal group. However, the decreased expression of sesn2 disrupted such a cellular defense mechanism by inhibiting AMPK-mediated antioxidative effects. As a result, LPS-induced molecular events could not be blocked in the sesn2 knockdown group, suggesting that sesn2 silencing might increase cellular sensitivity to LPS.

## 5. Conclusion

**Figure 6:**
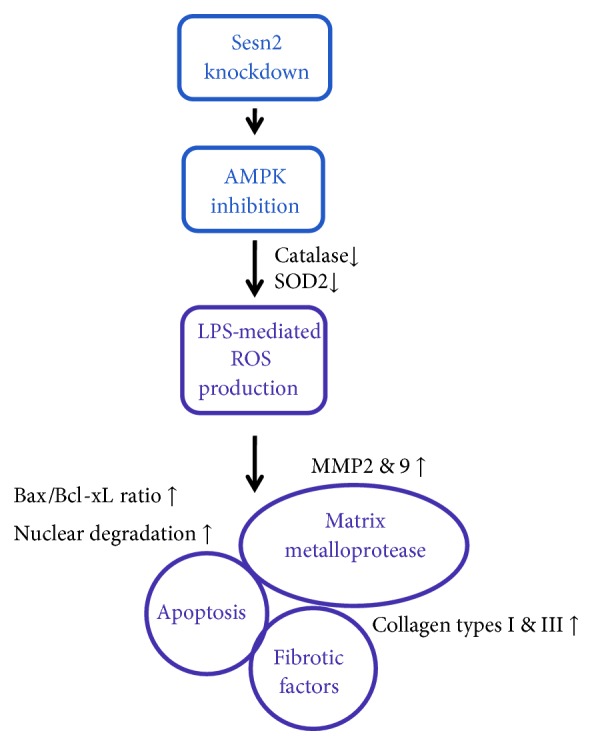
Schematic diagram of sesn2 functions in cardiac tissue. Sesn2 knockdown increases LPS-mediated oxidative stress, apoptosis, and fibrotic reactions in cardiomyocytes by inhibiting AMPK phosphorylation.

We report here for the first time that sesn2 regulates the effects of LPS treatment in H9c2 cells and C57BL/6 mice. Sesn2 knockdown strongly increased LPS-mediated ROS production by reducing the expression of antioxidant genes, including catalase and SOD2, thereby enhancing apoptotic signaling. Furthermore, LPS-mediated expression of MMP-2, MMP-9, collagen type I, and collagen type III was significantly increased in sesn2 knockdown heart tissues. These effects were significantly attenuated by treatment with an AMPK activator. In conclusion, inhibition of sesn2 aggravated LPS-induced oxidative, apoptotic, and fibrotic reactions by inhibiting AMPK phosphorylation (Figure 6), suggesting that sesn2 is a novel potential therapeutic target for preventing the progression of cardiomyopathy-related molecular events.

## Abbreviations

AICAR: 5-Aminoimidazole-4-carboxamide ribonucleotide
AMPK: 5′ AMP-activated protein kinase
ANOVA: Analysis of variance
Bax: Bcl2-associated X protein
Bcl-xL: B-cell lymphoma-extra large
DAPI: 4′,6-Diamidino-2-phenylindole
DHE: Dihydroethidium
H_2_O_2_: Hydrogen peroxide
LPS: Lipopolysaccharide
MMP: Matrix metalloproteinase
ROS: Reactive oxygen species
Sesn2: Sestrin2
shRNA: Short hairpin RNA
SOD2: Superoxide dismutase 2
TLR4: Toll-like receptor 4
TUNEL: Terminal deoxynucleotidyl transferase dUTP nick end labeling.
