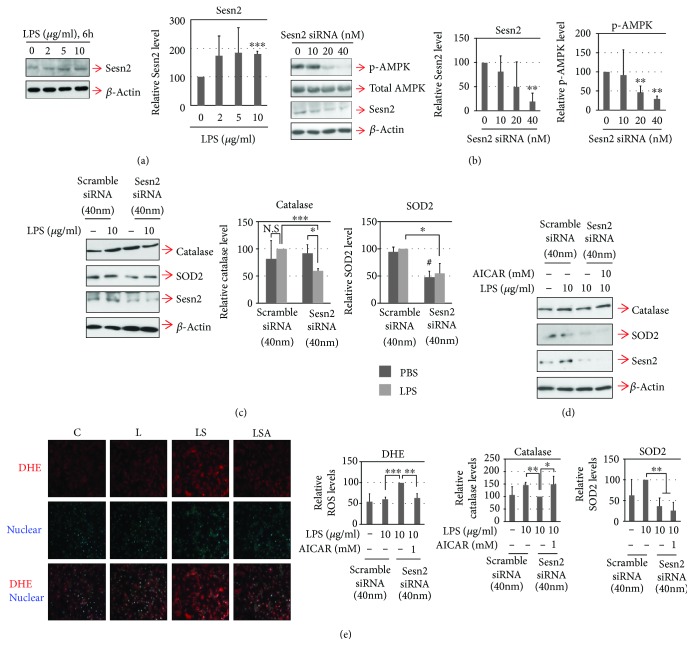# Erratum to “Knockdown of Sestrin2 Increases Lipopolysaccharide-Induced Oxidative Stress, Apoptosis, and Fibrotic Reactions in H9c2 Cells and Heart Tissues of Mice via an AMPK-Dependent Mechanism”

**DOI:** 10.1155/2019/1905313

**Published:** 2019-02-03

**Authors:** Hwan-Jin Hwang, Joo Won Kim, Hye Soo Chung, Ji A. Seo, Sin Gon Kim, Nan Hee Kim, Kyung Mook Choi, Sei Hyun Baik, Hye Jin Yoo

**Affiliations:** Division of Endocrinology and Metabolism, Department of Internal Medicine, College of Medicine, Korea University, Seoul, Republic of Korea

In the article titled “Knockdown of Sestrin2 Increases Lipopolysaccharide-Induced Oxidative Stress, Apoptosis, and Fibrotic Reactions in H9c2 Cells and Heart Tissues of Mice via an AMPK-Dependent Mechanism” [1], the western blots in Figures 1(b) and 1(d) are the same. This happened during the production process and the publisher apologizes to the authors and readers for this error. The corrected version of Figure 1 is shown below.

## Figures and Tables

**Figure 1:**